# The Impact of Aerobic Dance Intervention on Postural Balance in Children: A Randomized Controlled Trial

**DOI:** 10.3390/children11050573

**Published:** 2024-05-10

**Authors:** Ghada Jouira, Cristina Ioana Alexe, Khawla Zinelabidine, Haithem Rebai, George Danuț Mocanu, Adin Marian Cojocaru, Luciana Dragomir, Denis Čaušević, Sonia Sahli

**Affiliations:** 1 Research Laboratory Education, Motricité, Sport et Santé (EM2S) LR19JS01, High Institute of Sport and Physical Education of Sfax, University of Sfax, Sfax 3029, Tunisia; jouiraghada0825@gmail.com (G.J.); khawlazinelabidine@gmail.com (K.Z.); sonia.sahli@isseps.usf.tn (S.S.); 2 Department of Physical Education and Sports Performance, “Vasile Alecsandri” University of Bacău, 600115 Bacău, Romania; 3 Tunisian Research Laboratory ‘Sports Performance Optimization’ (LR09SEP01), National Center of Medicine and Science in Sports (CNMSS), Tunis 1002, Tunisia; haithem.rebai@yahoo.fr; 4 Individual Sports and Physical Therapy Department, “Dunărea de Jos” University of Galati, 800008 Galați, Romania; 5 Faculty of Physical Education and Sport, Spiru Haret University, 041905 Bucharest, Romania; ushefs_cojocaru.adin@spiruharet.ro; 6 Doctoral School of Accounting, Bucharest University of Economic Studies, 010374 Bucharest, Romania; dragomirluciana23@stud.ase.ro; 7 Faculty of Sport and Physical Education, University of Sarajevo, 71000 Sarajevo, Bosnia and Herzegovina; denis.causevic@fasto.unsa.ba

**Keywords:** postural balance, school children, dance, physical activity, vision

## Abstract

This study aimed to investigate the impact of an 8-week aerobic dance intervention on postural balance in children. Forty-one children, aged 9 to 11, were randomly assigned to either an aerobic dance group (ADG) or a control group (CG) from a primary school. Postural balance was assessed using center of pressure (CoP) excursions before and after the 8-week intervention period. Evaluations were conducted on both firm and foam surfaces in bipedal and unipedal stances under open-eyes (OE) and closed-eyes (CE) conditions, as well as on both medial–lateral (ML) and anterior–posterior (AP) surfaces in a bipedal stance under OE conditions. The ADG exhibited significantly decreased CoP_Vm_ values during firm bipedal CE, unipedal OE, foam bipedal OE and CE, and foam unipedal OE (*p* < 0.005). This study suggests that aerobic dance intervention improved postural balance in children, showcasing adaptability and improved stability under various conditions.

## 1. Introduction

Achieving and maintaining postural balance is a crucial element of human physiological function, involving a complex interplay between sensory input and motor responses. The coordination of subconscious reflex reactions, particularly involving visual, proprioceptive, and vestibular systems, is fundamental for achieving optimal postural balance [1]. This equilibrium serves as a foundational element for upright posture, coordinated movements, and successful adaptation to environmental changes, playing a crucial role in facilitating engagement in daily activities and basic motor skills [2,3].

The developmental journey, particularly from the first to the seventh year of life [4,5,6], with some researchers extending this period to the eighth or ninth year [7], emerges as a crucial phase. During this period, there is a pivotal emphasis on neuromuscular coordination and sensory integration, establishing a solid foundation for postural balance [8]. The subsequent period introduces a distinct phase marked by neurohormonal changes, temporarily impairing postural control due to the intricate interaction between hormonal fluctuations and the continuous maturation of physiological systems [8,9]. This transitional period witnesses a gradual decline in the function of the balance system, persisting until approximately the thirtieth year of life [10]. Consequently, interventions aimed at maintaining or enhancing postural balance during this critical period are crucial.

In the field of pediatric postural balance research, special emphasis is placed on studying children as they progress through the various stages of development. This focus is important due to the significant changes in postural control that occur during childhood and adolescence. Physical activity is widely recognized for its role in enhancing physical fitness [11,12]. The current research places a significant emphasis on comprehending how physical activity influences postural balance during various developmental stages. Indeed, engaging in physical activity stimulates sensory information integration, strengthening the connection between visual, proprioceptive, and vestibular systems [13,14], ultimately refining motor responses and improving postural balance [15]. It has been found that regular physical activity interventions positively affect postural balance in children aged between 7 and 13 [16]. In addition, better physical fitness in children and adolescents correlates with improved postural balance [17]. Another study also found that active children, compared to their sedentary counterparts, exhibit superior postural balance, suggesting that physical activity contributes to its efficient development [18].

Aerobic dance, a motivating physical activity that has gained popularity in recent decades, stands out as a promising avenue [19]. An aerobic dance program is not only easily designed by physical education teachers but is also generally enjoyable for students, promising to foster intrinsic motivation and enhance the enjoyment of physical education classes [20]. This multisensory training requires participants to integrate various sensory cues to position their bodies in space [21], making it potentially effective for improving postural balance in children. The complex choreography, involving sagittal and straddling steps, engages multiple muscle groups, requires rhythmic coordination, and can be sustained for at least 10 min, in which balance, locomotion, and agility are needed [22]. A plyometric lunge was included with the expectation of improving muscle strength in the lower extremities [23,24]. The inclusion of a plyometric lunge aims to improve lower extremity muscle strength [22,23]. This enhancement, particularly in the lower extremities, within the aerobic dance program holds the potential for the development of stabilizing muscles, fostering greater stability and control during various postural tasks. 

Previous studies on aerobic dance in children and adolescents have demonstrated its potential to positively impact various aspects. Improvements have been demonstrated in various aspects, including body image dissatisfaction, executive functions, stress levels, motivation, self-esteem, academics, behavior, and social skills, following aerobic dance interventions [20,25,26,27,28,29,30]. Some studies noted limitations, such as short intervention durations [25] and reliance on self-report measures [20,25,28]. In addition, while these studies have provided valuable knowledge about the psychological and cognitive benefits of aerobic dance interventions, there remains a notable gap in the literature regarding their effects on physical outcomes, particularly postural balance. Understanding the influence of aerobic dance on postural balance is crucial, especially for children in their developmental stages, as it plays a significant role in their overall physical well-being and motor development. While previous intervention studies have demonstrated aerobic dance’s capacity to actively control body posture and enhance core stability, primarily in older individuals [31,32,33], there is a surprising lack of research focusing on its effects on postural balance in children. Several studies have delved into various dance programs and their impact on children’s postural balance. One study integrated sports dance into fitness training, resulting in improvements in posture, muscle strength, and balance [34]. Another study focused on creative dance, revealing enhancements in proprioception [35]. Similarly, ballet training was associated with improved proprioception, postural balance, and rhythmic synchronization [36,37]. Furthermore, a modern dance program enhanced both static and dynamic balance in girls [38]. Despite these findings, no study has yet explored the effects of aerobic dance on children’s postural balance.

Investigating the impact of aerobic dance on postural balance in children, particularly under various sensory manipulations, such as stances, eye conditions, and surface types, is of significant importance. Bipedal and unipedal stances are fundamental in evaluating postural balance, with unipedal stance imposing greater postural demands and requiring enhanced neuromuscular control [39]. Moreover, open- and closed-eyes conditions are essential in examining the influence of visual feedback on postural balance, with open open-eyes (OE) condition allowing individuals to use visual cues to maintain balance and closed-eyes (CE) condition challenging individuals to rely more on proprioceptive and vestibular inputs [40,41]. Furthermore, different surface types, such as firm, foam, and seesaw surfaces, present varying levels of stability. While firm surfaces provide a stable surface, foam surfaces introduce instability, requiring individuals to make finer adjustments in weight distribution and joint movements to stabilize [42]. Also, seesaw surfaces further challenge individuals by introducing dynamic movements that necessitate continuous adjustments in response to shifting weight distribution [43]. Systematically varying sensory conditions allows this study to discern the effects of aerobic dance on the relationship between sensory inputs and motor responses during postural control tasks. Understanding how these factors interact can provide information regarding the potential benefits of aerobic dance as an intervention for enhancing postural balance in this age group.

This study contributes to our understanding of the benefits of aerobic dance on physical outcomes, particularly postural balance, in children. The findings have broader implications for physical education programs, highlighting the importance of integrating dynamic and rhythmic activities. These activities not only enhance enjoyment, motivation, and pleasure but also promote motor skill development and improve postural balance. By fostering a positive attitude towards physical activity from an early age, aerobic dance interventions can help instill lifelong habits of physical fitness and promote a healthy lifestyle. Therefore, this study aims to investigate the impact of an aerobic dance intervention on postural balance in children. We hypothesize that participation in the aerobic dance program will lead to significant enhancements in postural balance under various sensory manipulations among this age group.

## 2. Materials and Methods

### 2.1. Participants

The sample size was calculated using the G*Power 3.1 software (Franz Faul-University of Kiel, Kiel, Germany) [44]. We set the statistical probability value or α at 0.05, statistical power at 0.80, and the non-sphericity correction = 1. Based on the previous literature [32] and discussions between the authors, we estimated the effect size for this research at 0.4. The initial sample size was calculated to be 46. 

The selection of participants underwent a meticulous three-step screening process. Initially, 47 children aged 9 to 11 from a primary school lacking scheduled physical education classes were enrolled. In the second stage, 43 children meeting specific inclusion and exclusion criteria were chosen. A pediatrician, applying Tanner’s (1962) criteria, categorized them as pre-pubertal (stage 1). Inclusion criteria were having a middle socio-economic status, which was determined using parents’ income, education level, and occupation; being free of physical or mental conditions that could impact pre-and post-testing; having no history of locomotor surgeries; the absence of respiratory dysfunctions; having no visual or auditory impairments; and having no cardiovascular or metabolic disorders. These details were gathered from the school’s enrollment database. In the final screening, two children were excluded for failing to meet pre-training measures or being absent during familiarization or pre-training test sessions. This resulted in a final study cohort of 41 children. Participants were randomly assigned to aerobic dance (ADG) and control (CG) groups (Table 1).

None of the participants reported engagement in structured physical activity or exercise programs, and all indicated a low level of physical activity based on responses to the Physical Activity Questionnaire for Children (PAQ-C) [45]. 

Following a comprehensive explanation of procedures, including potential risks and benefits, children provided their assent, and their parents or legal guardians provided written informed consent. This study was conducted with adherence to the principles of the Declaration of Helsinki and received approval from the local ethics committee.

### 2.2. Study Design

This study was designed as a randomized controlled training intervention, in which participants were systematically assigned to either the ADG or the CG. Throughout the intervention, the ADG underwent an 8-week aerobic dance program integrated into their physical education classes at school. This program, which was carefully conducted by an aerobic dance instructor with extensive experience, aimed to enhance participants’ physical fitness and coordination. Simultaneously, the CG refrained from engaging in any physical education or structured physical training, continuing with their regular daily activities throughout the same 8-week period. All aerobic dance sessions for the ADG were supervised by a qualified instructor specializing in aerobic dance, ensuring the safety and proper execution of exercises. The sessions followed a thoughtfully structured sequence of increasing difficulty levels to minimize the risk of injuries. To maintain the experimental conditions, participants across both groups were explicitly instructed not to engage in any physical activity during the entire duration of the experiment. Participants underwent a familiarization session with all the study tests and procedures two days before the experimental protocol began. The familiarization with CoP sway measurements lasted about 10 min to allow the participant to try all postural balance conditions with 1–2 min of rest. 

### 2.3. Intervention

The aerobic dance program for the ADG took place over 8 weeks, including two 45 min sessions per week. These sessions were carried out in a well-equipped aerobics room, included dance exercises of moderate to vigorous intensity, and were aligned with specific objectives detailed in Table 2. Morning sessions took place between 9:00 a.m. and 9:45 a.m., starting with a standardized rhythm and a 10 min warm-up. This warm-up involved simple movements of the upper and lower limbs, as well as stretching exercises, maintaining consistency throughout the training period. The core of each aerobic session consisted of 30 min of continuous aerobic dance exercises, divided into three separate dance situations lasting 10 min each. After the main exercise routine, there was a 5 min recovery period, including relaxation exercises accompanied by slow, rhythmic music. During these sessions, participants learned four new aerobic dance steps, gradually building a small routine. At the end of each session, this routine was repeated two or three times without interruption, serving to both reinforce and assess choreographic learning. Music played a central role in modulating exercise intensity. The warm-up phase featured music with an intensity of 100 beats per minute. During the first six weeks of the program, the intensity of the main exercise varied between 60% and 75% of the maximum heart rate, with the accompanying music set at 125–130 beats per minute. Subsequently, from weeks seven to fourteen, the intensity increased to 75 to 85 percent of the maximum heart rate, with music set at 135 to 145 beats per minute. Over the past two weeks, the intensity has gradually decreased to 60 to 70 percent of the maximum heart rate, with music set to 130 beats per minute. The entire tempo of the music was meticulously controlled using a CD player equipped with a speed controller.

### 2.4. Measurements

Participants were asked to maintain their static postural balance barefoot as much as possible on a static stabilometric platform (PostureWin© Techno Concept^®^, Cereste, France; 40 Hz frequency, 12-bit A/D conversion) in two postural conditions. In the bipedal stance condition, the participants’ legs were straight, and the (30°) configurations were suggested as feet position with the heels slightly apart (3 to 5 cm). In the unipedal stance condition, participants stood on their dominant leg, which was used to kick a ball placed on the floor in front of them. Participants stood on the platform under three different surface conditions, including a firm surface (the rigid surface of the force platform), a foam surface (surface consisted of a foam block [466 mm length × 467 mm width × 134 mm height above ground] with a density of 21.3 kg/m^3^ and an elastic modulus of 20.900 N/m^2^ mounted on the rigid surface of the force platform) [42], and a seesaw surface (radius 55 cm and arrow of 6 cm, positioned on the platform either in the ML or AP directions) [46]. The CoP sways of each participant were recorded under the following conditions: firm bipedal-OE, firm bipedal-CE, firm unipedal-OE, firm unipedal-CE, foam bipedal-OE, foam bipedal-CE, foam unipedal OE, ML seesaw bipedal OE, and AP seesaw bipedal OE. The order of the postural conditions was counterbalanced. The participants were instructed to refrain from using their arms for balance during the recording sessions and to keep them alongside their bodies. The experimenter was present to ensure security, but no additional assistance was provided. Following the French Posturology Association norms, the duration of each trial was standardized to 25.6 s. A total of three trials was conducted for each experimental condition, and trial invalidation only occurred if there was a complete loss of balance. The best of three trials was analyzed to obtain a representative measure of postural behavior. To prevent fatigue, a 1 min resting interval was included between trials, during which participants were required to sit comfortably in a chair.

The selected parameter to evaluate the participants’ postural balance was the CoP mean velocity (CoP_Vm_). The CoP_Vm_, expressed in mm/s, represented the sum of the accumulated CoP displacement divided by the total time of the measurement. This parameter reflects the efficiency of the postural control system and is considered the measurement with the greatest reliability among trials [47].

### 2.5. Statistical Analysis

Data were analyzed using the program SPSS 25.0 (Statistical Package for the Social Sciences Inc., Chicago, IL, USA). The 95% confidence interval (CI) was calculated for means. Following the confirmation of data normality through the Shapiro–Wilk test, a series of mixed-model repeated measures ANOVAs were conducted to assess the impact of various factors on CoP_Vm_ values. The assumption of sphericity was confirmed for each ANOVA using Mauchly’s test. The initial four-way mixed-model repeated measures ANOVA (Group × Stance × Vision × Intervention) was employed to investigate the influence of group (ADG/CG), stance (bipedal/unipedal), vision (OE/CE), and intervention (Before/After) factors on the CoP_Vm_ values. Moreover, a four-way mixed-model repeated measures ANOVA (group × surface × vision × intervention) examined the impact of group (ADG/CG), surface (firm/foam), vision (OE/CE), and intervention (before/after) on the CoP_Vm_ values. Furthermore, a three-way mixed-model repeated measures ANOVA (group × surface × intervention) was used to assess the effect of group (ADG/CG), surface (firm/foam/seesaw ML/seesaw AP), and intervention (before/after) on the CoP_Vm_ values. A four-way mixed-model repeated measures ANOVA (group × surface × stance × intervention) investigated the influence of group (ADG/CG), surface (firm/foam), vision (OE/CE), stance (bipedal/unipedal), and intervention (before/after) on the CoP_Vm_ values. To determine whether the statistically significant differences found were practically significant, the effect size of each outcome measure was calculated. The partial eta squared (ηp^2^) formula was calculated for the main effects and interactions (small: 0.01 < ηp^2^ < 0.06; moderate: 0.06 < ηp^2^ < 0.14; large: ηp^2^ > 0.14), and the Cohen’s d was calculated for the pairwise differences (trivial: d < 0.2; small: 0.2 ≤ d < 0.5; moderate: 0.5 ≤ d < 0.8; large: d ≥ 0.8) [48]. Bonferroni adjustment for multiple comparisons was conducted. The alpha level of statistical significance was set at *p* < 0.05.

## 3. Results

In The initial four-way mixed-model repeated measures ANOVA revealed a significant main effect for the stance (F_(1,39)_ = 723.79; *p* < 0.001; ηp^2^ = 0.94), vision (F_(1,39)_ = 408.00; *p* < 0.001; ηp^2^ = 0.91), and intervention (F_(1,39)_ = 12.03; *p* = 0.001; ηp^2^ = 0.23) factors. However, no significant effect was observed for the group factor. The only interactions that reached significance were intervention × group (F(_1,39)_ = 5.63; *p* = 0.02; ηp^2^ = 0.12), stance × vision (F_(1,39)_ = 228.13; *p* < 0.001; ηp^2^ = 0.85), and stance × intervention (F_(1,39)_ = 5.74; *p* = 0.02; ηp^2^ = 0.12). The results of the second four-way mixed-model repeated measures ANOVA indicated a significant main effect for surface (F_(1,39)_ = 111.63; *p* < 0.001; ηp_2_ = 0.74), vision (F_(1,39)_ = 209.03; *p* < 0.001; ηp^2^ = 0.84), and intervention (F_(1,39)_ = 15.02; *p* < 0.001; ηp^2^ = 0.27), but no significant effect for the group factor. The only significant interactions were intervention × group (F_(1,39)_ = 6.50; *p* = 0.01; ηp^2^ = 0.14), intervention × surface (F_(1,39)_ = 4.42; *p* = 0.042; ηp^2^ = 0.10), and vision × surface (F_(1,39)_ = 52.11; *p* < 0.001; ηp^2^ = 0.57). The three-way mixed-model repeated measures ANOVA (group × surface × intervention) revealed a significant main effect for the surface (F_(1,37)_ = 538.38; *p* < 0.001; ηp^2^ = 0.97) and intervention (F_(1,39)_ = 25.58; *p* < 0.001; ηp^2^ = 0.40) factors, but no significant effect for the group factor. The interactions that reached significance were intervention × group (F(_1,39)_ = 16.26; *p* < 0.001; ηp^2^ = 0.29), intervention × surface (F_(1,37)_ = 6.83; *p* = 0.001; ηp^2^ = 0.35), and intervention × surface × (F_(1,37)_ = 4.41; *p* = 0.009; ηp^2^ = 0.26). The analysis of the last four-way mixed-model repeated measures ANOVA revealed a significant main effect for the surface (F_(1,39)_ = 911.00; *p* < 0.001; ηp^2^ = 0.95), stance (F_(1,39)_ = 1235.00; *p* < 0.001; ηp^2^ = 0.96), and intervention (F_(1,39)_ = 18.74; *p* < 0.001; ηp^2^ = 0.32) factors, but no significant effect for the group factor. The interactions that reached significance were intervention × group (F_(1,39)_ = 16.65; *p* < 0.001; ηp^2^ = 0.29), intervention × stance (F_(1,39)_ = 7.6; *p* = 0.009; ηp^2^ = 0.16), surface × stance (F_(1,39) =_ 436; *p* < 0.001; ηp^2^ = 0.91), and intervention × stance × group (F_(1,37)_ = 6.8; *p* = 0.013; ηp^2^ = 0.14).

Before the training intervention, the Bonferroni test did not reveal any statistically significant differences between the ADG and CG on the CoP_Vm_ values. After the training intervention, the Bonferroni test demonstrated statistically significant lower CoP_Vm_ values for the ADG compared to the CG only in foam bipedal OE, firm unipedal OE, and seesaw ML bipedal OE (See Table 3).

**Figure 1:**
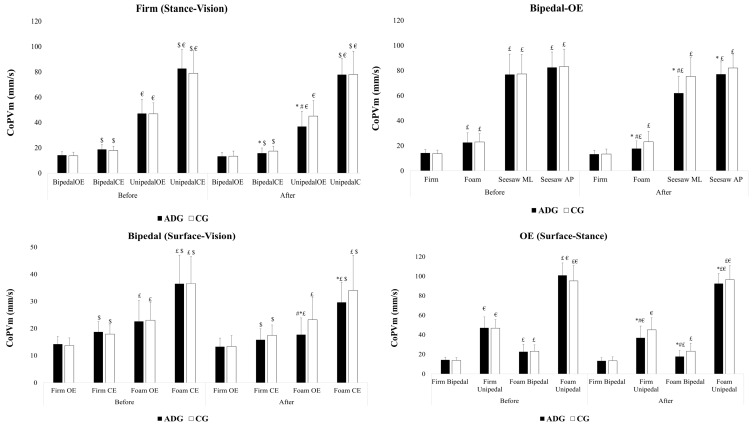
CoP_Vm_ values between ADG and CG in firm bipedal-open eyes (OE), firm bipedal-closed eyes (CE), firm unipedal-OE, firm unipedal-CE, foam bipedal-OE, foam bipedal-CE, foam unipedal OE, medial–lateral (ML) seesaw bipedal OE and anterior–posterior (AP) seesaw bipedal OE conditions before and after the aerobic dance intervention. Notes: *: Significant difference (*p* < 0.005) between before and after; ^#^: Significant difference (*p* < 0.05) between ADG and CG; ^$^: Significant difference (*p* < 0.001) between OE and CE conditions; ^€^: Significant difference (*p* < 0.001) between bipedal and unipedal; ^£^: Significant difference (*p* < 0.001) between firm and foam or ML seesaw or AP seesaw.

In the ADG, the Bonferroni test revealed a significant decrease in CoP_Vm_ values after compared to before the training intervention, specifically in firm bipedal CE, firm unipedal OE, foam bipedal OE, foam bipedal CE, seesaw ML bipedal OE and in the seesaw AP bipedal OE (see Table 3, Figure 1). No significant difference was observed in the CG.

Before and after the training intervention, the Bonferroni test revealed that visual removal significantly increased the CoP_Vm_ values under both the firm bipedal and unipedal conditions in the ADG (bipedal/before: *p* = 0.001, d = 1.34; bipedal/after: *p* = 0.04, d = 0.69; unipedal/before: *p* < 0.001, d = 2.66; unipedal/after *p* ≤ 0.001, d = 3.15) and in the CG (bipedal/before: *p* < 0.001, d = 1.35; bipedal/after: *p* ≤ 0.001, d =1.02; unipedal/before: *p* < 0.001, d = 2.28, unipedal/after: *p* ≤ 0.001, d = 2.10), as well as under the foam bipedal condition in both the ADG (before: *p* < 0.001, d = 1.49; after: *p* < 0.001, d = 1.67) and CG (before: *p* < 0.001, d = 1.59; after: *p* < 0.001, d = 1.10) (Figure 1).

The Bonferroni test indicated that the CoP_Vm_ values significantly increased in the firm unipedal compared to firm bipedal stances in the ADG under both the OE (before: *p* < 0.001, d = 4.03; after: *p* < 0.001, d = 2.57) and CE (before: *p* < 0.001, d = 5.76; after: *p* < 0.001, d = 6.45) conditions and in the CG under both the OE (before: *p* < 0.001, d = 5.12; after: *p* < 0.001, d = 3.46) and CE (before: *p* < 0.001, d = 4.75; after: *p* < 0.001, d = 4.44) conditions, as well as in the foam unipedal OE stance compared to the foam bipedal OE stance in the ADG (before: *p* < 0.001, d = 7.42; after: *p* < 0.001, d = 8.74) and CG (before: *p* < 0.001, d = 5.99; after: *p* < 0.001, d = 6.37) (Figure 1).

Additionally, the Bonferroni test revealed that the CoP_Vm_ values significantly increased in the foam bipedal in the ADG under the OE (before: *p* < 0.001, d = 1.44; after: *p* < 0.001, d = 1.03) and CE (before: *p* < 0.001, d = 2.22; after: *p* < 0.001, d = 2.30) conditions and in CG under the OE (before: *p* < 0.001, d = 1.84; after: *p* < 0.001, d = 1.46) and CE (before: *p* < 0.001, d = 2.49; after: *p* ≤ 0.001, d = 1.73) conditions and in the seesaw ML bipedal stance in the ADG (before: *p* < 0.001, d = 5.36; after: *p* < 0.001, d = 5.05) and CG (before: *p* < 0.001, d = 5.66; after: *p* < 0.001, d = 5.65), as well as in the seesaw AP bipedal stance in the ADG (before: *p* < 0.001, d = 7.64; after: *p* < 0.001, d = 7.68) and CG (before: *p* < 0.001, d = 6.90; after: *p* < 0.001, d = 7.67) (Figure 1).

## 4. Discussion

The objective of this study was to explore the impact of an aerobic dance intervention on postural balance in children. Postural balance, a crucial aspect of human physiological function, relies on the relationship between sensory inputs and motor responses. The coordination of subconscious reflex reactions, mediated by the visual, proprioceptive, and vestibular systems, forms the basis for maintaining an upright posture, executing coordinated movements, and adapting to environmental changes [49]. The investigation of specific interventions, such as aerobic dance, becomes essential for understanding their influence on postural balance, particularly during critical developmental phases.

This study’s results showed a decrease in CoP_Vm_ values after the intervention, within the ADG across various postural balance conditions. This decrease indicates an improvement in postural balance after the aerobic dance intervention, particularly in challenging conditions. Our findings align with prior studies indicating that various forms of dance, such as aerobic dance, contribute to postural balance enhancement in different populations [22,50,51,52].

Within the ADG, participants demonstrated a decrease in CoP_Vm_ values under the firm bipedal CE condition. These findings suggest that individuals who underwent aerobic dance training exhibited an enhanced ability to maintain postural balance, even in the absence of visual cues. This improvement highlights the adaptability of sensory systems, especially proprioception and vestibular function. Proprioception, which governs the body’s awareness of its position in space [53], and vestibular function, which is responsible for detecting head movements and spatial orientation, are pivotal for maintaining balance [54]. Indeed, aerobic dance routines, which are characterized by dynamic and rhythmic sequences, demand continuous adjustments in response to changing positions and sequences. This constant need for adaptation stimulates the central nervous system to refine its ability to process and integrate sensory information from sources such as vision, proprioception, and the vestibular system [55,56]. One key aspect of this adaptation is the enhancement of proprioceptive awareness [57]. The diverse choreography and weight-shifting movements inherent in aerobic dance prompt participants to develop a heightened sense of the relative positions of their body parts [57]. This heightened proprioception allows for more accurate adjustments in weight distribution and limb positioning, contributing to improved postural adjustments, particularly in scenarios in which visual cues are limited [57]. In addition, aerobic dance often involves movements that stimulate the vestibular system [58], which is responsible for detecting head movements and spatial orientation. The varied surfaces, spins, turns, and dynamic shifts in body orientation challenge participants’ vestibular function [58,59,60]. This suggests that aerobic dance training may induce adaptive changes in the vestibular system, increasing sensitivity to head movements and improving spatial orientation. This, in turn, contributes to more effective postural responses and balance. Moreover, the dynamic and varied nature of aerobic dance routines provides continuous balance challenges that demand constant adjustments in muscle activity and joint coordination [61,62]. Regular participation in such activities likely enhances neuromuscular coordination, improving the ability to respond to perturbations and ultimately contributing to a more stable postural balance. The observed improvements in postural balance following aerobic dance training can be seen as a manifestation of neuroplasticity [63,64], reflecting the brain’s capacity to adapt and reorganize itself in response to sensory input. Dance, as a sensorimotor activity, stimulates multiple layers of the neural system, including those involved in motor planning and execution, sensory integration, and cognitive processing. This stimulation leads to enhanced functional connectivity between the basal ganglia, cerebellum, and prefrontal cortex [65]. The multifaceted sensory demands of aerobic dance likely trigger neuroplastic changes [21] that optimize the efficiency of sensory processing and motor responses, further refining postural balance.

However, the lack of a significant difference under the firm unipedal conditions with CE may suggest that aerobic dance training might not have had a substantial impact on postural balance in the absence of visual input on challenging stance. This result could be influenced by the intricate interplay between proprioceptive and vestibular mechanisms, with visual input potentially playing a more dominant role in this specific challenging condition (unipedal, CE). It is important to note that in the context of firm bipedal with OE, when participants were standing on a firm surface with both feet and their eyes open, there was a lack of change in the CoP_Vm_ values following the aerobic dance intervention. This may imply that under these particular conditions, the intervention did not induce a measurable improvement in postural balance. These results underscore the specificity of the aerobic dance training effects on postural balance, highlighting improvements in certain sensory conditions while revealing potential limitations in others. These findings contribute to a more nuanced understanding of how aerobic dance interventions influence postural balance under varying sensory challenges.

The observed improvement in postural balance under foam bipedal OE and CE and foam unipedal OE conditions in the ADG following an 8-week intervention suggests the efficacy of aerobic dance training in enhancing stability on an unstable surface. The decrease in CoP_Vm_ values implies improved balance on an unstable surface, reflecting the benefits of aerobic dance training in challenging sensory environments. Indeed, aerobic dance likely promotes greater joint proprioception [66,67], leading to increased joint position sense and better awareness of limb movements. This heightened proprioceptive acuity facilitates more accurate adjustments in response to the dynamic and unpredictable nature of the dance routines [68], contributing to enhanced postural stability on an unstable surface. Furthermore, the improved postural balance on an unstable surface after aerobic dance training may be due to the development of anticipatory postural adjustments [69,70]. In fact, the nature of aerobic dance, with its dynamic and rhythmic sequences, necessitates participants to anticipate and prepare for upcoming movements. A study on synchronized activity in the prefrontal cortex during anticipation of visuomotor processing found that synchronized oscillatory networks in the prefrontal cortex are involved in top-down anticipatory mechanisms that facilitate subsequent processing [71]. This suggests that anticipation likely stimulates the prefrontal cortex, leading to enhanced planning and execution of postural adjustments in anticipation, further refining postural balance. The cognitive processes involved in anticipating and responding to the next dance step may translate to improved predictive postural balance, allowing individuals to better adapt to the challenges posed by an unstable surface. Furthermore, the continuous sensory-motor challenges inherent in aerobic dance may contribute to the refinement of the sensorimotor integration process [72], in which the brain becomes more adept at processing and using sensory information for effective motor responses, ultimately enhancing postural balance on unstable surfaces, like a foam surface.

The results of this study showed a significant decrease in CoP_Vm_ values within the ADG after the training intervention under the ML and AP seesaw in a bipedal stance with OE condition. This suggests an improvement in postural balance during seesaw movements in both side-to-side (ML) and front-to-back (AP) directions. The improvement in postural balance during these dynamic movements indicates a more refined and adaptable response to the demands of the dance intervention, reflecting the nuanced adjustments in postural control and balance. This aligns with the idea that dance, especially aerobic dance, or dance in general, can lead to improved proprioception, enhanced postural stability, and a more sophisticated response to dynamic and challenging movement patterns, ultimately contributing to better postural control and balance [68,73,74,75,76]. Moreover, the rhythmic and coordinated nature of aerobic dance routines requires participants to engage multiple muscle groups simultaneously, promoting overall muscle strength and endurance [32,62]. This muscular development contributes to greater stability during challenging movements [77]. In addition, the constant changes in direction and speed inherent in aerobic dance routines challenge the vestibular system [32,78], which plays a key role in balance and spatial orientation. This can challenge the body to adapt quickly to different stimuli, improving proprioception, coordination, and postural balance in challenging dynamic movements such as ML and AP seesaw bipedal conditions [79].

The result of this study revealed lower CoP_Vm_ values in the OE compared to the CE condition, regardless of group, intervention, stance, or surface factors. The results indicate that visual information plays a primary role in influencing postural control, and the lack of visual feedback disrupts the mechanisms involved in maintaining balance. In addition, when transitioning from a bipedal stance to a more challenging unipedal stance or from a firm to a foam surface, significant increases in CoP_Vm_ values were observed, regardless of the group or intervention factors. These elevated CoP_Vm_ values suggest that individuals faced greater difficulties in maintaining postural balance under these challenging postural conditions compared to the relatively easier task of balancing on both legs in the firm bipedal condition [80]. The results emphasize the important role of postural and surface conditions in influencing postural balance in this population. Indeed, when individuals shift from a stable bipedal stance to a unipedal stance or to standing on a foam surface, the demands on their postural control system increase significantly [80,81]. 

It is essential to acknowledge certain limitations in this study. While our study demonstrated the positive impact of 8 weeks of aerobic dance on children’s postural balance, further exploration into the lasting effects of such interventions is needed. Longitudinal studies could assess whether improvements in postural balance observed during the intervention period persist over time and whether they could potentially reduce the risk of falls or injuries while also enhancing physical performance in various activities. Additionally, investigating the potential long-term effects of aerobic dance interventions lasting beyond 8 weeks may reveal even greater benefits. Moreover, comparing the effects of aerobic dance with those of other physical activities, such as traditional sports, could provide valuable information into the most effective training modalities for promoting postural balance and overall physical development in children. Furthermore, expanding the demographic scope of future studies to include a more diverse range of participants in terms of age, ethnicity, socio-economic status, and health profiles would enhance the generalizability of the findings. As well, incorporating more diverse physical conditions and a larger sample size could offer a more comprehensive understanding of the diverse benefits of aerobic dance. In addition, this study did not directly investigate the underlying mechanisms responsible for the observed improvements in postural balance following aerobic dance training. Future studies should incorporate additional methodologies, such as electromyography, or other physiological measures, to delve into the intricate mechanisms involved in the neuroplastic changes, sensory adaptations, and motor responses associated with aerobic dance training. Another limitation of our study is the absence of formal tools specifically designed to objectively evaluate exercise safety and execution. While our aerobic dance sessions were supervised by a qualified instructor with extensive experience, the use of standardized assessment tools could have provided additional objective measures of safety and effectiveness. Incorporating such tools in future studies would enhance the robustness of our findings and provide additional assurance regarding exercise safety and effectiveness.

### Practical Implications

The present study aimed to explore the impact of an aerobic dance intervention on postural balance in children, focusing on the relationship between sensory inputs and postural responses. The results demonstrated significant improvements in postural balance following the intervention, particularly under challenging conditions, such as unipedal stances, foam surfaces, and dynamic seesaw movements. The theoretical implications of our findings lie in elucidating the mechanisms underlying the observed improvements in postural balance. Engaging in dynamic and rhythmic activities likely induced neuroplastic changes that optimized sensory processing and motor coordination, leading to enhanced postural balance. These findings contribute to our understanding of how sensorimotor activities like aerobic dance modulate neural pathways involved in postural control in children. Moreover, our study offers significant practical implications for educators and healthcare professionals. By demonstrating the adaptability of sensory systems and the effectiveness of aerobic dance in enhancing postural balance, our findings underscore the importance of incorporating such activities into physical education programs. This adaptability is significant in real-life situations in which children may encounter diverse and unpredictable environmental conditions, promoting motor skill development and enhancing postural balance. Healthcare professionals can also use aerobic dance interventions in clinical settings to address postural balance problems in children.

## 5. Conclusions

In conclusion, this study aimed to investigate the impact of aerobic dance intervention on postural balance in children. The findings revealed a significant decrease in CoP_Vm_ values within ADG, particularly in challenging conditions. The ADG showed decreased CoP_Vm_ values under firm bipedal CE, unipedal OE, foam bipedal OE and CE, and foam unipedal OE conditions, indicating enhanced postural balance. In seesaw movements, both in the ML and AP directions, the ADG exhibited decreased CoP_Vm_ values, indicating refined postural balance during dynamic movements. These results emphasize the positive impact of aerobic dance on postural balance in children aged between 9 and 11, showcasing adaptability and improved stability under various conditions.

## Figures and Tables

**Table 1:** Participant characteristics, expressed as means and SD.

	ADG	CG	Degree of Freedom	Independent *t*-Test
	N = 19 (10 Boys, 9 Girls)	N = 22 (12 Boys, 10 Girls)		
Age (years)	10.3 ± 0.57	10.03 ± 0.43	39	*p* = 0.07
Height (cm)	138.85 ± 7.07	139.33 ± 7.73	39	*p* = 0.09
Weight (kg)	38.67 ± 7.89	37.55 ± 7.81	39	*p* = 0.83
BMI (kg/m^2^)	17.37 ± 3.09	19.20 ± 4.54	39	*p* = 0.15

Abbreviations: ADG: aerobic dance group; CG: control group; BMI: body mass index.

**Table 2:** Session objectives for the aerobic dance training program.

Session No.	Training Objectives
1 + 2	- To develop a detailed understanding of rhythm across diverse beats per minute (BPM) and dance styles. - To identify and comprehend the structural organization of a dance block (4 × 8 counts), with particular attention to identifying downbeat accents.
3 + 4	- To recognize the initiation and conclusion points of dance phrases while maintaining a consistent rhythmic cadence. - To master the execution of walking steps within the tempo range of 125–130 BPM, focusing on precise foot placement and rhythm.
5	- To proficiently execute Sequence A, encompassing specific steps such as Marching, Mambo, V Step, and Basic Step. - To memorize step sequences and associated cues at 125–130 BPM, ensuring accurate synchronization of footwork with the music.
6	- To replicate Sequence B, consisting of movements like forward slap and lunge, to reinforce motor learning and coordination.
7	- To perform Sequence C, featuring steps like step touch and heel step, to further enhance motor coordination and execution proficiency.
8	- To engage in collaborative group work aimed at designing and executing a cohesive sequence of 4 basic step blocks at a tempo range of 135–145 BPM, emphasizing collective synchronization and cohesion in performance.
9	- To execute a series of 2 basic step blocks for each movement at 135–145 BPM, demonstrating accurate recall of steps and cues while maintaining synchronization with the music tempo.
10	- To repeat the performance of a sequence of 2 basic step blocks for each movement at 135–145 BPM to consolidate motor skills and proficiency.
11	- To design and execute an intergroup sequence comprising 2 blocks at 135–145 BPM, focusing on seamless transitions and coordination between participating groups.
12	- To integrate previously learned sequences into a unified performance block, incorporating arm movements at 135–145 BPM to enhance expressiveness and fluidity in movement execution.
13 + 14	- To innovate and perform a novel single-block sequence featuring diverse basic steps at 135–145 BPM, demonstrating creativity and adaptability in choreography. - To ensure synchronization with the designated music tempo throughout the performance.
15	- To prepare for upcoming performances by rehearsing formations at 130 BPM, emphasizing spatial awareness and precision in alignment within the performance space.
16	- To execute a concise dance routine comprising two blocks at 130 BPM, focusing on maintaining consistent footwork precision and rhythmic synchronization throughout the performance.
Note	1 block = 32 counts; BPM = beats per minute; 2 × 8 = two aerobic dance sentences (16 counts)

**Table 3:** Comparative analysis of center of pressure mean velocity values before and after the training intervention in aerobic dance group (ADG) and control group (CG) under visual (open and closed eyes (OE, CE)), postural (bipedal, unipedal), and surface (firm, foam) conditions.

Groups	ADG		CG							
	means ± SD, (95% CI)		means ± SD, (95% CI)		ADG Before/After		CG Before/After		After ADG/CG	
CoP_Vm_ (mm/s)	Before	After	Before	After	*p*	d	*p*	d	*p*	d
Firm Bipedal OE	14.19 ± 2.82 (12.83 to 15.56)	13.26 ± 3.16 (11.74 to 14.79)	13.70 ± 2.79 (12.46 to 14.94)	13.36 ± 4.03 (11.57 to 15.15)	=0.33	-	=0.70	-	=0.47	-
Firm Bipedal CE	18.71 ± 3.81 (16.87 to 20.55)	15.79 ± 4.07 (13.83 to 17.76)	17.90 ± 3.40 (16.39 to 19.41)	17.41 ± 3.87 (15.69 to 19.12)	=0.001	=0.74	=0.50	-	=0.20	-
Firm Unipedal OE	47.12 ± 11.20 (41.72 to 52.52)	37.20 ± 12.74 (31.06 to 43.34)	46.81 ± 8.70 (42.95 to 50.67)	45.07 ± 12.30 (39.61 to 50.53)	<0.001	=0.82	=0.43	-	=0.03	=0.62
Firm Unipedal CE	82.68 ± 15.15 (75.38 to 89.98)	77.81 ± 12.97 (71.56 to 84.07)	78.92 ± 17.84 (71.01 to 86.83)	77.93 ± 18.39 (69.78 to 86.09)	=0.16	-	=0.76	-	=0.98	-
Foam Bipedal OE	22.60 ± 7.72 (18.88 to 26.32)	18.26 ± 6.04 (15.37 to 21.20)	23.04 ± 6.61 (20.11 to 25.97)	22.29 ± 7.59 (18.92 to 25.66)	=0.003	=0.62	=0.90	-	=0.02	=0.59
Foam Bipedal CE	36.44 ± 10.60 (31.32 to 41.55)	29.61 ± 7.43 (26.02 to 33.19)	36.56 ± 10.03 (32.11 to 41.01)	34.03 ± 12.96 (28.28 to 39.78)	=0.004	=0.75	=0.23	-	=0.19	
Foam Unipedal OE	100.89 ± 12.75 (94.74 to 107.04)	92.48 ± 10.38 (87.47 to 97.49)	95.28 ± 15.72 (88.31 to 102.25)	96.48 ± 14.59 (90.00 to 102.95)	=0.001	=0.73	=0.60	-	=0.22	
Seesaw ML Bipedal OE	76.89 ± 16.28 (69.03 to 84.74)	62.01 ± 13.24 (55.62 to 68.39)	77.33 ± 15.64 (70.39 to 84.26)	75.37 ± 14.97 (68.73 to 82.01)	<0.001	=1.00	=0.40	-	=0.005	=0.94
Seesaw AP Bipedal OE	82.50 ± 12.32 (76.55 to 88.44)	77.08 ± 9.90 (72.31 to 81.86)	83.10 ± 13.93 (76.92 to 89.28)	82.05 ± 10.45 (77.14 to 86.68)	=0.004	=0.49	=0.52	-	=0.12	-

## Data Availability

The data that support the findings of this study are available on request from the corresponding author. The data are not publicly available due to privacy or ethical restrictions.

## References

[1] Orendorz-Frączkowska K., Kubacka M. (2020). The development of postural control in 6–17 old years healthy children. Part I Postural control evaluation in modified Clinical Test for The Sensory Interaction on Balance in 6–17 old year children (mctsib). Pol. J. Otolaryngol..

[2] Ghanbarzadeh A., Azadian E., Majlesi M., Jafarnezhadgero A.A., Akrami M. (2021). Effects of Task Demands on Postural Control in Children of Different Ages: A Cross-Sectional Study. Appl. Sci..

[3] Karlsson A., Frykberg G. (2000). Correlations between force plate measures for assessment of balance. Clin. Biomech..

[4] Nougier V., Bard C., Fleury M., Teasdale N. (1997). Contribution of central and peripheral vision to the regulation of stance. Gait Posture.

[5] Panaet E., Zwierzchoska A., Peyré-Tartaruga L., Alexe D., Rosołek B., Alexe C. (2023). Distribution of plantar pressures under static conditions, in various areas of the pediatric flatfoot in sensitive period of development–pilot study. Balneo PRM Res. J..

[6] Panaet A.E., Alexe C.I., Stângaciu O.A., Hazar F., Rață G., Alexe D.I., Hofmeister M. (2022). The effects of pediatric flat foot on the frontal alignment of proximal segment. Balneo PRM Res. J..

[7] Roncesvalles M.N., Schmitz C., Zedka M., Assaiante C., Woollacott M. (2005). From egocentric to exocentric spatial orientation: Development of posture control in bimanual and trunk inclination tasks. J. Mot. Behav..

[8] Stanek E., Truszczyńska A., Drzał-Grabiec J., Tarnowski A. (2015). Postural balance assessment in children aged 7 to 9 years, as related to body weight, height, and physical activity. Biomed. Hum. Kinet..

[9] John C., Rahlf A.L., Hamacher D., Zech A. (2019). Influence of biological maturity on static and dynamic postural control among male youth soccer players. Gait Posture.

[10] Drzał-Grabiec J., Snela S., Rykała J., Podgórska J., Banaś A. (2013). Changes in the body posture of women occurring with age. BMC Geriatr..

[11] Sunda M., Gilic B., Sekulic D., Matic R., Drid P., Alexe D.I., Cucui G.G., Lupu G.S. (2022). Out-of-School Sports Participation Is Positively Associated with Physical Literacy, but What about Physical Education? A Cross-Sectional Gender-Stratified Analysis during the COVID-19 Pandemic among High-School Adolescents. Children.

[12] Dobbins M., Husson H., DeCorby K., LaRocca R.L. (2013). School-based physical activity programs for promoting physical activity and fitness in children and adolescents aged 6 to 18. Cochrane Database Syst. Rev..

[13] Wallman-Jones A., Perakakis P., Tsakiris M., Schmidt M. (2021). Physical activity and interoceptive processing: Theoretical considerations for future research. Int. J. Psychophysiol..

[14] Jouira G., Rebai H., Alexe D.I., Sahli S. (2024). Postural Balance in Boys With Intellectual Disabilities Who Participate in Soccer Training. Pediatr. Exerc. Sci..

[15] Paillard T. (2023). The optimal method for improving postural balance in healthy young and older people: Specific training for postural tasks encountered in personal physical practice. Front. Physiol..

[16] Oliveira Junior E.d., Silva A.F.M.d., Antunes F.D., Jacinto J.L., Aguiar A.F. (2021). Analysis of postural balance in children who practice and those who do not practice sports activities. Rev. Bras. Med. Esporte.

[17] Bürgi F., Meyer U., Granacher U., Schindler C., Marques-Vidal P., Kriemler S., Puder J.J. (2011). Relationship of physical activity with motor skills, aerobic fitness and body fat in preschool children: A cross-sectional and longitudinal study (Ballabeina). Int. J. Obes..

[18] García-Soidán J.L., García-Liñeira J., Leirós-Rodríguez R., Soto-Rodríguez A. (2020). Physical activity practice and optimal development of postural control in school children: Are they related?. J. Clin. Med..

[19] Iermakov S., Podrigalo L., Alekseev A., Rovnaya O. (2016). Studying of interconnections of morphological functional indicators of students, who practice martial arts. Phys. Educ. Stud..

[20] Rokka S., Kouli O., Bebetsos E., Goulimaris D., Mavridis G. (2019). Effect of Dance Aerobic Programs on Intrinsic Motivation and Perceived Task Climate in Secondary School Students. Int. J. Instr..

[21] Rehfeld K., Lüders A., Hökelmann A., Lessmann V., Kaufmann J., Brigadski T., Müller P., Müller N.G. (2018). Dance training is superior to repetitive physical exercise in inducing brain plasticity in the elderly. PLoS ONE.

[22] Shigematsu R., Chang M., Yabushita N., Sakai T., Nakagaichi M., Nho H., Tanaka K. (2002). Dance-based aerobic exercise may improve indices of falling risk in older women. Age Ageing.

[23] Witzke K.A., Snow C.M. (2000). Effects of polymetric jump training on bone mass in adolescent girls. Med. Sci. Sports Exerc..

[24] Jouira G., Rebai H., Alexe D.I., Sahli S. (2024). Effect of Combined Training with Balance, Strength, and Plyometrics on Physical Performance in Male Sprint Athletes with Intellectual Disabilities. Adapt. Phys. Act. Q..

[25] Burgess G., Grogan S., Burwitz L. (2006). Effects of a 6-week aerobic dance intervention on body image and physical self-perceptions in adolescent girls. Body Image.

[26] Zinelabidine K., Elghoul Y., Jouira G., Sahli S. (2022). The effect of an 8-week aerobic dance program on executive function in children. Percept. Mot. Ski..

[27] Padia K., Sabuwala M., Dave N., Shaikh F., Bhatia D. (2017). Effect of Dance Aerobics on Perceived Stress in Physiotherapy Students. World J. Pharm. Res..

[28] Gao Z., Zhang T., Stodden D. (2013). Children’s physical activity levels and psychological correlates in interactive dance versus aerobic dance. J. Sport Health Sci..

[29] Marino D.M. (2010). Effects of Aerobic Dance on Self-Esteem, Academics, Behavior, and Social Skills. Ph.D. Thesis.

[30] Pelclová J., Frömel K., Skalik K., Stratton G. (2008). Dance and aerobic dance in physical education lessons: The influence of the student’s role on physical activity in girls. Acta Univ. Palacki. Olomuc. Gymnica.

[31] Hofgaard J., Ermidis G., Mohr M. (2019). Effects of a 6-week Faroese chain dance programme on postural balance, physical function, and health profile in elderly subjects: A pilot study. BioMed Res. Int..

[32] Wang L., Guo F., Zhao C., Zhao M., Zhao C., Guo J., Zhang L., Zhang L., Zhu W. (2023). The effect of aerobic dancing on physical fitness and cognitive function in older adults during the COVID-19 pandemic-a natural experiment. Sports Med. Health Sci..

[33] Bunyaratavej N., Kritpet T., Sukkeaw W. (2015). A comparison between the effects of aerobic dance training on mini-trampoline and hard wooden surface on bone resorption, health-related physical fitness, balance, and foot plantar pressure in Thai working women. J. Med. Assoc. Thail..

[34] Andrieieva O., Kashuba V., Yarmak O., Cheverda A., Dobrodub E., Zakharina A. (2021). Efficiency of children’s fitness training program with elements of sport dances in improving balance, strength and posture. J. Phys. Educ. Sport.

[35] Chatzopoulos D., Doganis G., Kollias I. (2018). Effects of creative dance on proprioception, rhythm and balance of preschool children. Early Child Dev. Care.

[36] Chatzopoulos D. (2019). Effects of Ballet Training on Proprioception, Balance, and Rhythmic Synchronization of Young Children. J. Exerc. Physiol. Online.

[37] Michalska J., Kamieniarz A., Fredyk A., Bacik B., Juras G., Słomka K.J. (2018). Effect of expertise in ballet dance on static and functional balance. Gait Posture.

[38] Marinkovic D., Belic A., Marijanac A., Martin-Wylie E., Madic D., Obradovic B. (2022). Static and dynamic postural stability of children girls engaged in modern dance. Eur. J. Sport Sci..

[39] Promsri A. (2022). Modulation of Lower-Limb Muscle Activity in Maintaining Unipedal Balance According to Surface Stability, Sway Direction, and Leg Dominance. Sports.

[40] Peterka R.J. (2018). Sensory integration for human balance control. Handb. Clin. Neurol..

[41] Gaerlan M.G., Alpert P.T., Cross C., Louis M., Kowalski S. (2012). Postural balance in young adults: The role of visual, vestibular and somatosensory systems. J. Am. Assoc. Nurse Pract..

[42] Patel M., Fransson P.-A., Lush D., Gomez S. (2008). The effect of foam surface properties on postural stability assessment while standing. Gait Posture.

[43] Almeida G., Carvalho R., Talis V. (2006). Postural strategy to keep balance on the seesaw. Gait Posture.

[44] Faul F., Erdfelder E., Lang A.-G., Buchner A. (2007). G*Power 3: A flexible statistical power analysis program for the social, behavioral, and biomedical sciences. Behav. Res. Methods.

[45] Crocker P., Bailey D.A., Faulkner R.A., Kowalski K.C., McGrath R. (1997). Measuring general levels of physical activity: Preliminary evidence for the Physical Activity Questionnaire for Older Children. Med. Sci. Sports Exerc..

[46] Jabnoun S., Borji R., Sahli S. (2019). Postural control of Parkour athletes compared to recreationally active subjects under different sensory manipulations: A pilot study. Eur. J. Sport Sci..

[47] Paillard T., Noé F. (2015). Techniques and methods for testing the postural function in healthy and pathological subjects. BioMed Res. Int..

[48] Cohen J. (2013). Statistical Power Analysis for the Behavioral Sciences.

[49] Horak F.B. (2006). Postural orientation and equilibrium: What do we need to know about neural control of balance to prevent falls?. Age Ageing.

[50] Coubard O.A., Ferrufino L., Nonaka T., Zelada O., Bril B., Dietrich G. (2014). One month of contemporary dance modulates fractal posture in aging. Front. Aging Neurosci..

[51] Salgado R., de Paula Vasconcelos L.A. (2010). The use of dance in the rehabilitation of a patient with multiple sclerosis. Am. J. Danc. Ther..

[52] Rodacki A.L.F., Cepeda C.P.C., Lodovico A., Ugrinowitsch C. (2017). The effects of a dance-based program on the postural control in older women. Top. Geriatr. Rehabil..

[53] Garcia-Falgueras A. (2016). An introduction to proprioception concept in Pilates and yoga. Br. J. Med. Med. Res..

[54] Angelaki D.E., Cullen K.E. (2008). Vestibular system: The many facets of a multimodal sense. Annu. Rev. Neurosci..

[55] Ladda A.M., Wallwork S.B., Lotze M. (2020). Multimodal sensory-spatial integration and retrieval of trained motor patterns for body coordination in musicians and dancers. Front. Psychol..

[56] Nigmatullina Y., Hellyer P.J., Nachev P., Sharp D.J., Seemungal B.M. (2015). The neuroanatomical correlates of training-related perceptuo-reflex uncoupling in dancers. Cereb. Cortex.

[57] Hutt K., Redding E. (2014). The effect of an eyes-closed dance-specific training program on dynamic balance in elite pre-professional ballet dancers: A randomized controlled pilot study. J. Danc. Med. Sci..

[58] Oliveira-Barreto A., Menezes P., Feitosa A., Oliveira P., Taguchi C., Passos P., Pereira L. (2017). Dancing effects on the magnitude of the vestibular-cervical reflex. Otolaryngol. Head Neck Surg..

[59] Anwar S., Zahid J., Alexe C.I., Ghazi A., Mareș G., Sheraz Z., Sanchez-Gomez R., Perveen W., Alexe D.I., Gasibat Q. (2024). Effects of Myofascial Release Technique along with Cognitive Behavior Therapy in University Students with Chronic Neck Pain and Forward Head Posture: A Randomized Clinical Trial. Behav. Sci..

[60] Fuchs D. (2018). Dancing with gravity—Why the sense of balance is (the) fundamental. Behav. Sci..

[61] Bobo M., Yarbrough M. (1999). The effects of long-term aerobic dance on agility and flexibility. J. Sports Med. Phys. Fit..

[62] Keogh J.W., Kilding A., Pidgeon P., Ashley L., Gillis D. (2009). Physical benefits of dancing for healthy older adults: A review. J. Aging Phys. Act..

[63] Müller P., Rehfeld K., Schmicker M., Hökelmann A., Dordevic M., Lessmann V., Brigadski T., Kaufmann J., Müller N.G. (2017). Evolution of neuroplasticity in response to physical activity in old age: The case for dancing. Front. Aging Neurosci..

[64] Leisman G., Aviv V. (2020). A neuroscience of dance: Potential for therapeusis in neurology. Brain and Art: From Aesthetics to Therapeutics.

[65] Meulenberg C.J., Rehfeld K., Jovanović S., Marusic U. (2023). Unleashing the potential of dance: A neuroplasticity-based approach bridging from older adults to Parkinson’s disease patients. Front. Aging Neurosci..

[66] Marmeleira J., Pereira C., Cruz-Ferreira A., Fretes V., Pisco R., Fernandes O. (2009). Creative dance can enhance proprioception in older adults. J. Sports Med. Phys. Fit..

[67] Wang H., Ji Z., Jiang G., Jiao X., Liu W. A Study on the Influence of Latin Dance and Tai Chi Exercise on Balance and Knee Joint Proprioception. Proceedings of the 2016 International Conference on Identification, Information and Knowledge in the Internet of Things (IIKI).

[68] Batson G. (2009). Update on proprioception: Considerations for dance education. J. Danc. Med. Sci..

[69] Strang A., Choi H., Berg W. (2008). The effect of exhausting aerobic exercise on the timing of anticipatory postural adjustments. J. Sports Med. Phys. Fit..

[70] Kanekar N., Aruin A.S. (2015). Improvement of anticipatory postural adjustments for balance control: Effect of a single training session. J. Electromyogr. Kinesiol..

[71] Liang H., Bressler S.L., Ding M., Truccolo W.A., Nakamura R. (2002). Synchronized activity in prefrontal cortex during anticipation of visuomotor processing. Neuroreport.

[72] Karpati F.J., Giacosa C., Foster N.E., Penhune V.B., Hyde K.L. (2016). Sensorimotor integration is enhanced in dancers and musicians. Exp. Brain Res..

[73] Granacher U., Muehlbauer T., Bridenbaugh S.A., Wolf M., Roth R., Gschwind Y., Wolf I., Mata R., Kressig R.W. (2012). Effects of a salsa dance training on balance and strength performance in older adults. Gerontology.

[74] Duncan C.A., Ingram T.G., Mansfield A., Byrne J.M., McIlroy W.E. (2016). Population differences in postural response strategy associated with exposure to a novel continuous perturbation stimuli: Would dancers have better balance on a boat?. PLoS ONE.

[75] Virtanen N., Tiippana K., Tervaniemi M., Poikonen H., Anttila E., Kaseva K. (2022). Exploring body consciousness of dancers, athletes, and lightly physically active adults. Sci. Rep..

[76] Kattenstroth J.-C., Kalisch T., Holt S., Tegenthoff M., Dinse H.R. (2013). Six months of dance intervention enhances postural, sensorimotor, and cognitive performance in elderly without affecting cardio-respiratory functions. Front. Aging Neurosci..

[77] Paillard T. (2017). Relationship between muscle function, muscle typology and postural performance according to different postural conditions in young and older adults. Front. Physiol..

[78] Swathi V., Sathish K.K. (2013). Influence of dance training on sacculocollic pathway: Vestibular evoked myogenic potentials (VEMP) as an objective tool. J. Evol. Med. Dent. Sci..

[79] Jouira G., Alexe C.I., Herlo J.N., Moraru C.E., Bogdan M., Alexe D.I., Mareș G., Sahli S. (2023). Effects of Smartphone Activities on Postural Balance in Adolescents with Intellectual Disabilities. Children.

[80] Jouira G., Srihi S., Kachouri H., Ben Waer F., Rebai H., Sahli S. (2021). Static postural balance between male athletes with intellectual disabilities and their sedentary peers: A comparative study. J. Appl. Res. Intellect. Disabil..

[81] Dingenen B., Staes F.F., Janssens L. (2013). A new method to analyze postural stability during a transition task from double-leg stance to single-leg stance. J. Biomech..

